# Neuronal Oscillations Enhance Stimulus Discrimination by Ensuring Action Potential Precision

**DOI:** 10.1371/journal.pbio.0040163

**Published:** 2006-05-16

**Authors:** Andreas T Schaefer, Kamilla Angelo, Hartwig Spors, Troy W Margrie

**Affiliations:** **1**Department of Physiology, University College London, London, United Kingdom; **2**WIN Research Group of Olfactory Dynamics, Max-Planck-Institut für medizinische Forschung, Heidelberg, Germany; Brown UniversityUnited States of America

## Abstract

Although oscillations in membrane potential are a prominent feature of sensory, motor, and cognitive function, their precise role in signal processing remains elusive. Here we show, using a combination of in vivo, in vitro, and theoretical approaches, that both synaptically and intrinsically generated membrane potential oscillations dramatically improve action potential (AP) precision by removing the membrane potential variance associated with jitter-accumulating trains of APs. This increased AP precision occurred irrespective of cell type and—at oscillation frequencies ranging from 3 to 65 Hz—permitted accurate discernment of up to 1,000 different stimuli. At low oscillation frequencies, stimulus discrimination showed a clear phase dependence whereby inputs arriving during the trough and the early rising phase of an oscillation cycle were most robustly discriminated. Thus, by ensuring AP precision, membrane potential oscillations dramatically enhance the discriminatory capabilities of individual neurons and networks of cells and provide one attractive explanation for their abundance in neurophysiological systems.

## Abstract 2

A combination of in vivo, in vitro, and theoretical approaches are used to demonstrate that membrane potential oscillations remove accumulating jitter in trains of action potentials.

## Introduction

Within neurons there exist various sources of membrane noise, including stochasticity of membrane conductances [ 1– 3], stimulus nonspecific synaptic conductances [ 4– 6], and variable synaptic transmission [ 7]. While noise sources may prove beneficial, for example, via stochastic resonance effects [ 8, 9], they generally limit action potential (AP) precision and thus the fidelity of communication between cells [ 2, 3]. This is supported by numerous experimental [ 10– 13] and theoretical [ 14– 16] studies that indicate that even for identical stimuli, the exact timing of AP discharge may differ substantially between trials. Spike output, however, is all the information a postsynaptic neuron has available to potentially discern specific patterns of activity occurring upstream in presynaptic cells. Are there mechanisms in place that might increase the robustness of spike discharge? Intrinsic events such as dendritic Na ^+^, Ca ^2+^, or *N*-methyl- d-aspartate (NMDA) spikes [ 17– 22] might reliably signal the presence of a particular type of event. Due to their all-or-none nature, they do not, however, readily permit the discrimination of more than a subset of stimuli. Furthermore, they appear insensitive to subtle stimulus-specific differences in the temporal properties of synaptic input patterns.

In the scenario where individual synaptic events are large enough to evoke spikes, it is well documented that specific patterns of large fast input waveforms are one means of producing temporally precise AP discharge [ 10, 13]. However, stimulus-evoked patterns of subthreshold activity often consist of a series of rather small temporally dispersed events occurring over a few to hundreds of milliseconds [ 5, 23, 24]. In most cells such inputs are typically processed in a highly nonlinear fashion in electrotonically dispersed dendritic locations and result in patterns of AP discharge that reflect stimulus-specific properties of the input and subsequent integrations performed within the cell [ 25]. Recently, it has been shown that in small, electrically compact cells that receive very few synaptic contacts, even individual quanta are capable of supporting reliable information transmission [ 23]. Most cell types, however, receive an extremely high number of synaptic inputs and a considerable fraction are needed to achieve threshold and encode a specific stimulus or motor command [ 5, 26– 28]. In such cases, many evoked currents must be integrated over time to produce AP discharge [ 28– 30]—the patterns of which are thought to represent a specific sensorimotor signal.

Membrane potential oscillations (MPOs) are a common feature of sensorimotor processing. They may be generated by synaptic and/or intrinsic mechanisms and have been attributed to synchronizing stimulus-relevant cell assemblies [ 31, 32] and providing a phase “tag” to individual spikes for efficient readout [ 33– 39]. In the hippocampus and the olfactory system, MPOs, at the individual cell level, are suggested to serve as an internal reference signal whereby spike trains could encode information by their phase relative to the background oscillation cycle [ 36, 37, 39].

Here we have tested the hypothesis that MPOs facilitate the discrimination of input patterns composed of small amplitude synaptic events. We show that MPOs ensure AP precision in the olfactory bulb in vivo *.* Using in vitro recordings, we subsequently describe the cellular mechanisms underlying the optimization of AP precision by MPOs and how this dramatically improves the discrimination of synaptic input patterns. Finally, using a variety of theoretical and in vitro approaches, we show that for a broad range of MPO properties, stimulus, and cellular parameters, ongoing oscillatory activity ensures near-perfect discrimination of temporally complex small amplitude synaptic input patterns.

## Results

### Synaptically Generated MPOs Enhance AP Precision In Vivo

**Figure 1:**
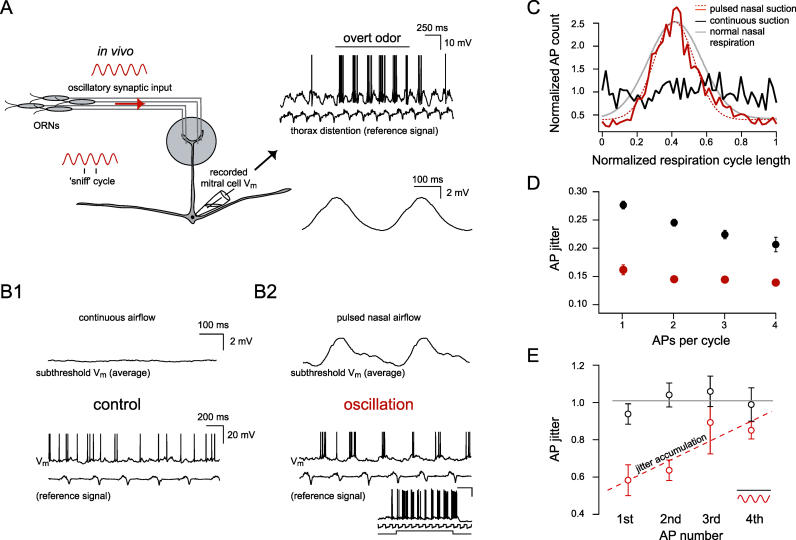
Synaptically Driven Oscillations Optimize AP Precision In Vivo (A) Schematic of the rodent olfactory receptor neuron (ORN) projection to a glomerulus in the olfactory bulb illustrating that oscillatory sensory input is coupled to the “sniff cycle” (inset, lower left). An example trace (top right) of a whole-cell recording from a representative mitral cell in a freely breathing mouse showing network-evoked oscillations in membrane potential due to background room-odor and overt odor (black line) stimulation. At the time indicated by the black horizontal bar, an overt odor stimulus (0.1% amyl acetate) evoked AP discharge. The simultaneously recorded thorax distention signal is shown directly underneath. Below an average trace is displayed, showing the subthreshold theta oscillation in the freely breathing preparation (traces averaged with respect to the thoracal breathing cycle [ 37]). (B1 and B2) Average of subthreshold voltage traces showing that the inherent oscillatory membrane potential in the freely breathing animal can be abolished and reproduced in a tracheotomized preparation. Below are single example traces showing that continuous airflow through the nose removes the MPO (B1), whereas pulsed nasal airflow (triggered by the thorax signal) mimics the subthreshold oscillatory drive and controls spike timing (B2). The inset shows an example of an overt odor-evoked response in the tracheotomized preparation. From top to bottom: membrane voltage recording (scale bars are 20 mV and 500 ms), nasal suction, and odor valve opening. Odor presented was 10% hexanol. In the constant airflow case, positive constant current was injected to evoke approximately the same number of APs per thoracal breathing cycle. (C) Overlapping histograms of AP times for continuous and pulsed airflow. Overlaid is the Gaussian fit of the pulsed airflow case (dashed red trace, *n* = 3 animals) and the freely breathing case ( *n* = 7, gray trace). (D) AP precision was determined by combining respiration cycles with the same number of APs and quantified by measuring the average distance of each AP to the mean AP time. The mean distance (“AP jitter”) is measured relative to the thorax signal and plotted as a function of the number of APs per cycle. Pooled data from *n* = 3 cells showing AP precision for the oscillation (red) and nonoscillation case (black). Lower values of the jitter indicate higher precision (plotted is the mean ± SEM). (E) AP precision data from the same experiments as in D, but normalized (gray lines) to the nonoscillation case for the first, second, third, and fourth AP within each oscillation cycle. Dashed line (red) is a linear fit to the oscillation data points. Note the jitter accumulation with AP number within an oscillation cycle for the synaptically driven oscillation.

A major limitation in determining the impact of oscillatory activity on signal processing in vivo has been the inability to record evoked activity in the controlled presence and absence of a natural oscillatory rhythm. In vivo experiments in mitral cells in the olfactory bulb of freely breathing mice reveal that the membrane potential is governed by a strong oscillatory synaptic rhythm ( Figure 1A) that is tightly coupled to the breathing cycle [ 37, 40– 42]. It is sensory-evoked by nasal airflow and thus sensitive to naris occlusion and blockers of excitatory synaptic transmission [ 37, 40, 41]. We directly measured the impact of the oscillatory rhythm on AP precision in vivo using a double tracheotomy approach [ 40] that mimics the oscillation observed in freely breathing animals ( Figure 1B1 and 1B2) while controlling overall excitability by injecting small amounts of constant current. Using the distention of the thorax as an external reference signal to track the oscillatory input [ 37, 42, 43], comparison of the normalized AP times for freely breathing and tracheotomized animals with pulsed nasal airflow confirms that both the sensory-evoked oscillations ( Figure 1A and 1B2) and overall AP distributions are indistinguishable in the two preparations (Kolmogorov-Smirnov, *p* > 0.05; *n* = 7 freely breathing, *n* = 3 tracheotomized; Figure 1C). This finding indicates a high degree of similarity between the oscillation-controlled AP patterning in the freely breathing and tracheotomized preparations. Importantly, this tracheotomy approach enables us to compare AP precision during sensory stimulation under oscillatory and nonoscillatory conditions in the presence of in vivo levels of membrane noise and background synaptic activity ( Figure 1B1 and 1B2).

By carefully controlling the overall, room odor–evoked AP rates via injection of depolarizing and/or hyperpolarizing current (in both the control and oscillation cases), we could compare AP precision. We found that the oscillatory drive greatly enhanced the overall precision of APs irrespective of how many APs were evoked within an oscillation cycle [F _(1,656)_ = 20.1, *p* < 0.001; *n* = 3 mice, Figure 1D]. However, the dependence of AP precision on the number of preceding APs was highly significant [F _(3,240)_ = 7, *p* < 0.001; *n* = 3 mice, Figure 1E] and specific to the oscillation [nonoscillation case: F _(3,91)_ = 2, *p* > 0.1; *n* = 3 mice, Figure 1E]. This indicates that oscillatory activity in vivo, although providing an overall enhancement of AP precision, results in precision decay or jitter accumulation with increasing AP number per oscillation cycle.

### Intrinsically Generated MPOs Enhance AP Precision In Vitro

**Figure 2:**
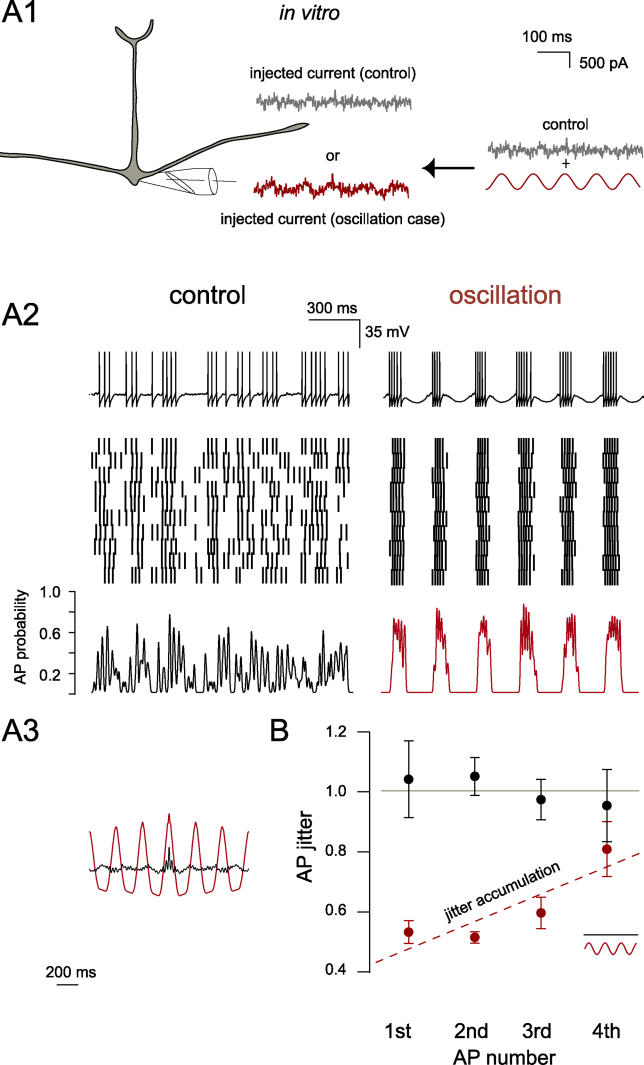
Current-Injection Evoked Oscillations Maintain Optimal AP Precision In Vitro (A1) Experimental configuration for examining the impact of oscillations on AP precision in vitro. Under control conditions, mitral cells recorded in vitro received input current injected via the pipette consisting of known Poisson trains convoluted with EPSC-like waveforms and added noise (see Materials and Methods). In the oscillation condition, a sine wave was added to the control stimulus. (A2) (Top) Single traces showing the voltage recorded under the two conditions in A1. Immediately below are rasters of AP discharge for ten repetitions (different random seeds for noise generation) for a single stimulus under control and oscillatory conditions. (Bottom) PSTH from the raster plots above. Spike trains were smoothed with a Gaussian filter (5 ms). (A3) Autocorrelation of the PSTHs shown in A2. (B) AP precision data from experiments as in A2, where AP jitter is normalized (gray line) to the nonoscillation case for the first, second, third, and fourth AP within each oscillation cycle. Dashed line (red) is a linear fit to the oscillation data points. Note that—as with the in vivo data ( Figure 1E)—AP jitter accumulates with AP number within an oscillation cycle.

The precision of a given AP is, in general, determined by two main factors: the noisy background intrinsic and synaptic currents, and the accumulating jitter of earlier spikes [ 10, 13]. To explore how oscillations achieve AP precision, we examined AP precision in olfactory bulb slices, a preparation that permits precise control of the input waveforms to mitral cells. In slices we not only can mimic background oscillatory drive by injecting sinusoidal current waveforms but also can quantitatively correlate injected input trains ( Figure 2A1) with patterned AP output.

It was crucial to first determine whether the general finding observed in vivo could be faithfully reproduced in the slice where noise and input sources can largely be exogenously controlled. We therefore injected stimulus patterns into mitral cells that consisted of a series of excitatory postsynaptic potential (EPSP) waveforms (containing noise) with and without oscillatory current ( Figure 2A1). We then compared the precision of AP trains within each oscillation period to those AP trains occurring within the same period under control conditions ( Figure 2A2, bottom). We found that oscillatory current injection not only resulted in an oscillatory peristimulus time histogram (PSTH) ( Figure 2A3) but also greatly enhanced the overall precision of APs. This enhancement was highly similar to that observed with synaptically generated oscillations in vivo (first AP: *p* < 0.05, *n* = 3 in vivo; *p* < 0.05, *n* = 7 in vitro; Figures 1E and 2B).

One obvious effect of the oscillation arises from compression or bundling of spikes around the cycle peak. However, this explanation does not account for the development of jitter across a single oscillation cycle. Despite spike train compression ( Figure 2A2 and 2A3), APs in the oscillation case deteriorated rapidly toward randomness as a function of the number of evoked APs within each cycle ( Figure 2B). This finding is in remarkable agreement with the synaptically generated in vivo data (fourth AP, *p* > 0.3; *n* = 3 in vivo, *n* = 7 in vitro; Figure 1E). Thus, in both the in vitro and in vivo preparations, we found that APs occurring during oscillation cycles are far more precise than under control conditions, with precision deteriorating as a function of AP number. Furthermore, “synaptic” and “intrinsic” (injected) oscillations produced quantitatively identical spike precision behavior.

### Mechanism of Oscillation-Mediated AP Precision

**Figure 3:**
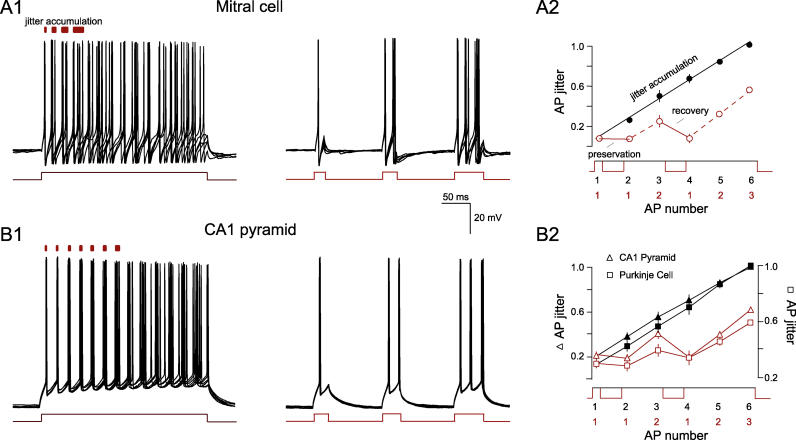
Membrane Potential Hyperpolarization Recovers AP Precision (A1) Five overlaid consecutive traces of the membrane voltage of a representative mitral cell in response to a long current injection that elicited an AP train of increasing imprecision. Red bars (above) indicate increasing AP jitter accumulation with time. (Right) Five voltage traces from the same cell where spiking is interrupted by 100-ms hyperpolarizing pulses, thus imitating oscillations with one, two, and three APs. Current pulse amplitude is 250 pA. (A2) The mean ± SEM of the AP jitter from recordings with (red) and without (black) intermittent hyperpolarizing pulses plotted against AP number. Each data point is normalized to the jitter of the sixth spike. AP precision is fully preserved or recovered by the hyperpolarizing intervals ( *n* = 11). (B1) Five overlaid consecutive traces of the membrane voltage of a representative CA1 pyramidal cell in response to a long current injection that elicited an AP train of increasing imprecision. Red bars (above) indicate increasing AP jitter accumulation with time. (Right) Five voltage traces from the same cell where spiking is interrupted by 100-ms hyperpolarizing pulses, thus imitating oscillations with one, two, and three APs. Current pulse amplitude is 300 pA. (B2) The mean ± SEM of the AP jitter from recordings with (red) and without (black) intermittent hyperpolarizing pulses plotted against AP number. Each data point is normalized to the jitter of the sixth spike. AP precision is fully preserved or recovered by the hyperpolarizing intervals (CA1 pyramids, triangles, *n* = 5, left axis; and Purkinje neurons, squares, *n* = 3, right axis).

The quantitative similarity between the in vivo and the in vitro data indicates that both the enhanced precision and its deterioration are cellular phenomena independent of any unique features of the in vivo environment. As such, oscillation-mediated enhancement of precision could occur in a variety of cell types and the mechanism of AP precision can be examined in the more controlled environment of the in vitro preparation. We propose that the *trough* of the oscillation removes AP jitter by ensuring an extended period without AP discharge, thus interrupting and preventing the accumulation of AP jitter. To test this hypothesis, we subjected cells to square depolarizing pulses interleaved by hyperpolarizing pulses, the simplest imitation of an in vivo oscillation ( Figure 3A1). Spiking periods of long duration evoked by depolarizing current steps induced AP jitter accumulation over time (0.07 ± 0.03 versus 0.26 ± 0.1, first versus second AP, *p* < 0.001, *n* = 11 cells; Figure 3A1 and 3A2) as observed previously across each individual oscillation cycle ( Figure 2B). Introducing hyperpolarizing intervals between the first and second APs fully preserved the precision of the following AP (0.07 ± 0.03 ms versus 0.07 ± 0.03 ms, *n* = 11, first versus next AP, *p* > 0.2; Figure 3A2). Furthermore, if AP jitter was permitted to accumulate over an AP train ( Figure 3A2), we found that the same level of hyperpolarization recovered 100% of AP precision (99 ± 5% recovery, *n* = 11; Figure 3A2). Since mitral cells are known to possess an unusual set of intrinsic conductances [ 44– 46], we repeated the jitter accumulation protocol in other principal cells ( Figure 3B), most notably Purkinje cells in the cerebellum ( Figure 3B2; *n* = 3) and CA1 pyramidal neurons of the hippocampus ( Figure 3B1 and 3B2; *n* = 5). In these experiments, we observed a virtually identical effect of hyperpolarization on AP precision recovery (98 ± 24% and 114 ± 6%, respectively; Figure 3B), indicating that oscillations improve AP precision irrespective of cell-specific intrinsic properties.

**Figure 4:**
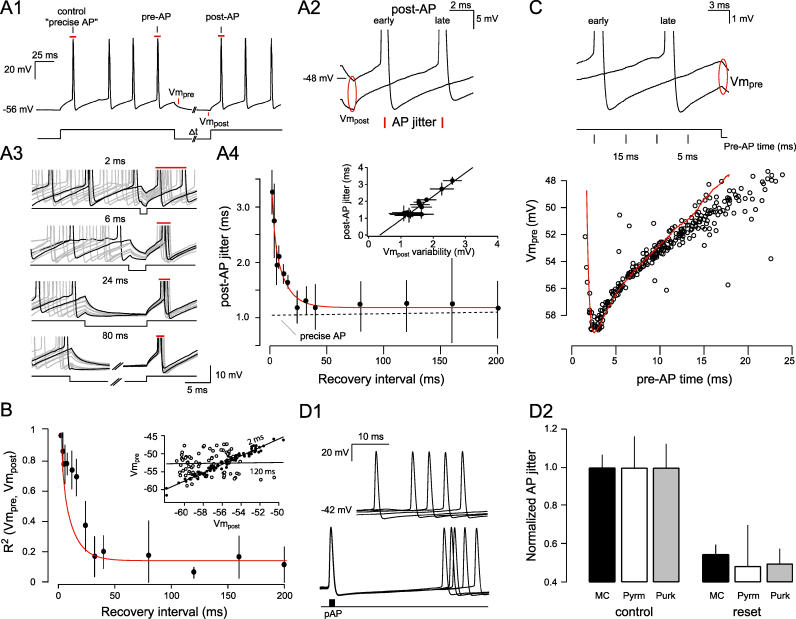
Hyperpolarizations Maintain AP Precision by Minimizing Membrane Potential Variance (A1) Schematic showing the experimental configuration and analysis parameters of the mitral cell membrane voltage recorded while varying the hyperpolarization period (Δt; 2 to 400 ms). AP jitter was created by injecting a depolarizing current pulse (150 to 250 pA for 100 ms) and estimated by calculating the standard deviation of the pre-AP time. The effect of the hyperpolarizing pulse on precision recovery was measured by determining the jitter of the AP immediately following the hyperpolarization pulse (post-AP) and comparing it to the control AP. The relationship between membrane potential at the beginning (Vm _pre_) and at the end (Vm _post_) was determined by calculating the mean voltage over the first 250 μs and the last 100 μs of the nonspiking interval. (A2) Two example traces of a 2-ms hyperpolarizing pulse with post-APs showing that depolarized (−48 mV) and hyperpolarized (−58 mV) potentials evoked early and late post-APs, respectively. The red ellipse highlights the variable membrane potential at the end of the hyperpolarizing pulse; red lines indicate the variable AP times of the post-AP that reflect post-AP jitter. (A3) Representative voltage traces of APs for hyperpolarization intervals of 2, 6, 24, and 80 ms show a large variation in Vm _pre_ (see also C); traces with the earliest and the latest prehyperpolarization AP are highlighted in black. Ten overlaid traces show the reduction in the variable membrane potential across the recovery period. The associated reduction in post-AP jitter is indicated by the red bars above the clipped APs. (A4) Post-AP jitter as a function of the recovery interval (mean ± SEM, *n* = 4 cells). The data points were fitted with a single exponential (τ = 6.8 ms). The precision of the control AP is indicated by the dashed line. Inset: Correlation between the post-AP jitter and membrane potential variance at Vm _post_ ( *R* ^2^ = 0.86). (B) The correlation between Vm _pre_ and Vm _post_ plotted as a function of the recovery interval ( *n* = 4). The graph is overlaid by the single exponential fit shown in A4 (red line). Inset: The Vm _pre_ and Vm _post_ values are plotted for a 2-ms interval (filled circles) and compared to that for a 120-ms interval (open circles). (C) (Top) Example traces showing the relationship between the pre-AP time (relative to the pulse onset) and Vm _pre_. (Below) A plot of Vm _pre_ against pre-AP time for a single cell. A single AHP trace is superimposed on the graph. (D1) Five consecutive traces from a mitral cell show spontaneous AP jitter relative to the same randomly chosen point in time (black) and the jitter of the AP (red) immediately following a precise AP (left). Cells were held at threshold by injecting constant current and the precise AP (pAP) was elicited by brief current injection (1,000 pA for 2 ms). (D2) Population data from mitral cells ( *n* = 6), pyramidal neurons ( *n* = 5), and Purkinje neurons ( *n* = 3) showing the normalized jitter of ongoing APs and the AP immediately following the injected pAP (mean ± SEM, *p* < 0.001 in all cell types). Precision recovery is similar in the three cell types.

To determine the minimum time necessary for full precision recovery, we allowed jitter to accumulate across spike trains and varied the duration of the hyperpolarizing pulse from 2 to 400 ms ( Figure 4A1). The timing of the first posthyperpolarization AP (“post-AP”; Figure 4A1) was calculated and found to be highly sensitive to the membrane potential at the end of the hyperpolarization period. Depolarized potentials elicited early spikes, and hyperpolarized potentials evoked late spikes ( *R* ^2^ = 0.66 ± 0.17, slope = −1.03 ± 0.15 ms/mV, *p* < 0.0005, *n* = 4; Figure 4A2). Thus, for brief hyperpolarization periods where the membrane potential remained highly variable, AP times were highly variable (correlation between jitter and posthyperpolarization membrane potential [Vm _post_] variability; *R* ^2^ = 0.86, *n* = 4; Figure 4A3 and 4A4, inset). Since longer hyperpolarization periods (greater than 20 ms) dramatically reduced the variance of Vm _post_, AP precision was reset to the levels of the most precise “control” AP [F _(13,39)_ = 8.94, *p* < 0.001, *n* = 4 cells; Figure 4A4]. In this way, membrane potential hyperpolarizations can ensure ongoing levels of “optimal” precision.

In Figure 4B we show the decorrelation between the membrane potential immediately prior to the hyperpolarization (Vm _pre_) and that following the hyperpolarization (Vm _post_). When compared to Figure 4A4, is evident that the time course of the decorrelation ( Figure 4B, black) closely matches the kinetics of precision recovery ( Figure 4B, red, and 4A4). Together, this indicates that cells exhibit a “memory” of the preceding membrane variance for approximately 20 ms and that the level of membrane potential variance at the end of the hyperpolarization determines the precision of the following AP. It further suggests that other “state” variables such as inactivation of channels or ion concentrations govern precision predominantly by influencing the membrane potential. To understand the factors contributing to such membrane potential variance, we examined the relationship between Vm _pre_ and preceding AP times. Figure 4C shows that the dynamics of this relationship reflects the kinetics of the afterhyperpolarization (AHP). This indicates that variable APs and their concomitant AHPs are the main source of membrane potential variance and AP jitter accumulation within spike trains. These conclusions are consistent with the observation that, within an oscillation cycle, spike trains become increasingly less precise when an intervening hyperpolarizing trough is absent ( Figure 2B).

If the overall level of AP precision relies on an ongoing reset mechanism, then it should be possible to recover some of the lost precision by injecting brief, large current transients to evoke a precise AP. Such a precise AP should provide the necessary reset to remove the accumulated AP jitter. Rather than assaying AP precision using step current injections from hyperpolarized potentials ( Figures 3 and 4A) we injected constant DC to maintain the cell at threshold and evoked a precise AP randomly with respect to the preceding AP time ( Figure 4D1). We found that this precise AP indeed acted in part as a reset switch; the next AP was significantly more precise than preceding APs (normalized jitter 1 ± 0.17 versus. 0.53 ± 0.14, *p* < 0.001, *n* = 6 mitral cells; Figure 4D2). Although the absolute level of precision recovery was lower than that observed for APs evoked by step current injections (i.e., instantaneous depolarizations, Figures 3 and 4A [ 10, 13]), this result was observed in all cell types examined ( Figure 4D2; *n* = 5 CA1 pyramids, *n* = 3 Purkinje cells) and suggests that MPOs ensure AP precision at least in part by abolishing the membrane potential variance associated with otherwise ongoing, compounding variable APs/AHPs.

### MPOs Permit Reliable Separation of Spike Trains

**Figure 5:**
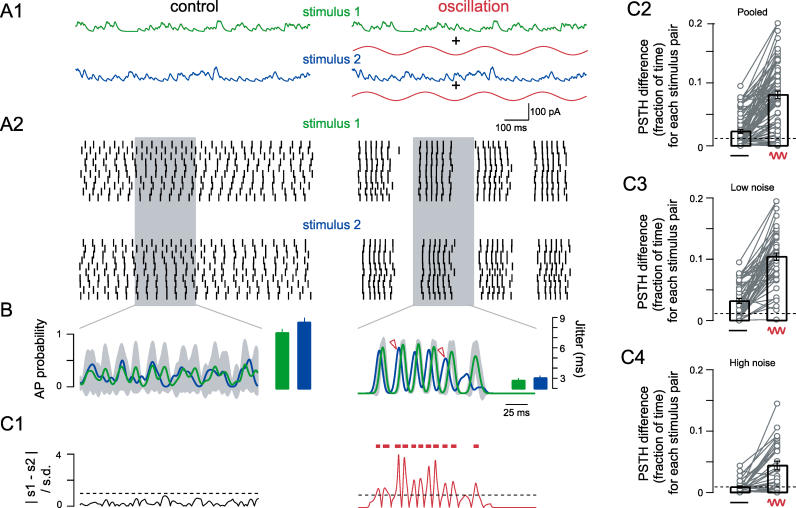
Oscillation-Mediated AP Precision Permits Separation of Stimulus-Specific Spike Trains In Vitro (A1) Stimuli (with the addition of randomly seeded noise, not shown) were evoked in CA1 pyramidal cells via the recording pipette in the absence and presence of an oscillation. (A2) Example raster plots show ten repetitions for two different stimuli with and without MPOs. (B) The normalized PSTH was calculated by averaging the ten smoothed (Gaussian filter; σ = 5 ms) spike trains shown in (A) and is plotted for stimulus 1 (green) and stimulus 2 (blue) separately (ordinate axis shown on left). Gray indicates the variance of the PSTH for stimulus 1 (SD of the ten [smoothed] repetitions shown in A2). Bar graphs show the overall AP precision for the entire stimulus period and both stimuli (ordinate axis far right). (C1) A PSTH difference plot showing the difference between the mean PSTHs corresponding to the two stimuli normalized to the variance (red bars indicate where the two stimulus-evoked spike trains can be separated because the normalized PSTH difference is larger than 1, dashed line). (C2) Population data showing temporal separation of all pairs of stimuli. Values given are the fraction of time the normalized PSTH difference was larger than 1 (e.g., red bars in C1, *n* = 7 cells, *n* = 77 stimulus pairs). (C3 and C4) PSTH differences separated based on the amplitude of injected noise (low σ = 0.1–0.2 mV, *n* = 3 cells, 48 stimulus pairs; high σ = 0.3–0.5 mV, *n* = 4 cells, 29 stimulus pairs). Dotted line indicates chance level.

The fundamental question arising from these data is whether neurons can make use of the improved precision that accompanies MPOs. We therefore next analyzed spike discharge patterns in response to two stimuli consisting of different trains of EPSP-like waveforms. The two stimuli differed only in the temporal arrangement of inputs ( Figure 5A1), not in average input rate or EPSP waveform. By “presenting” two stimuli to an individual CA1 pyramidal cell and repeating each stimulus ten times under different, randomly seeded background noise conditions (as in Figure 2), output spike trains can be compared in the presence and absence of the background MPO ( Figure 5A2). We compared the spike times across the entire stimulus period for repetitions of different stimuli in the absence and presence of an oscillation. Again, we found that the overall AP precision is strongly improved in the oscillation case ( Figure 5B, *p* < 10 ^−5^). To investigate whether the two stimuli result in distinguishable discharge patterns, we compared the resulting PSTHs ( Figure 5B). In the nonoscillation case, the PSTHs for both stimuli are relatively flat and variable, consistent with low precision (indicated by the large standard deviation exemplified for stimulus 1, gray shading in Figure 5B). The PSTH for stimulus 2 (blue) is consistently overlapping the PSTH for stimulus 1 (within the shaded area demarking one standard deviation of the PSTH for green stimulus 1). Thus, the difference between the two PSTHs is always smaller than the SD ( Figure 5C1, left), making it impossible to separate the two stimuli. In contrast, the presence of an MPO results in clear, distinct peaks, indicative of the high degree of precision ( Figure 5B, right). More important, AP timing is clearly different for the two stimuli as seen by the PSTHs that do not overlap during long stimulation epochs (e.g., arrowheads in Figure 5B, right). During these epochs (bars in Figure 5C1, right), the difference between the two PSTHs is substantially larger than the variance, making stimulus separation possible ( Figure 5C1, right). Thus, the fraction of time where the two PSTHs are significantly different indicates how readily two stimuli can be distinguished (termed “PSTH difference”).

We repeated this analysis for seven neurons and a total of 77 stimulus pairs. In all cases, MPOs dramatically increased the PSTH difference ( Figure 5C2, *p* < 5 × 10 ^−17^, *n* = 7 cells, *n* = 77 stimulus pairs). This observation was independent of the magnitude of injected and intrinsic noise ( Figure 5C3 and 5C4 low noise, *p* < 5 × 10 ^−14^; high noise, *p* < 5 × 10 ^−5^). Furthermore, in these experiments the parameters of the MPO remained constant. Since the two stimuli differed only in the temporal sequence of EPSPs, we can thus conclude that—through enhancing AP precision—MPOs allow for the discernment of different, temporally complex input patterns.

### MPOs Ensure High-Fidelity Pattern Discrimination

**Figure 6:**
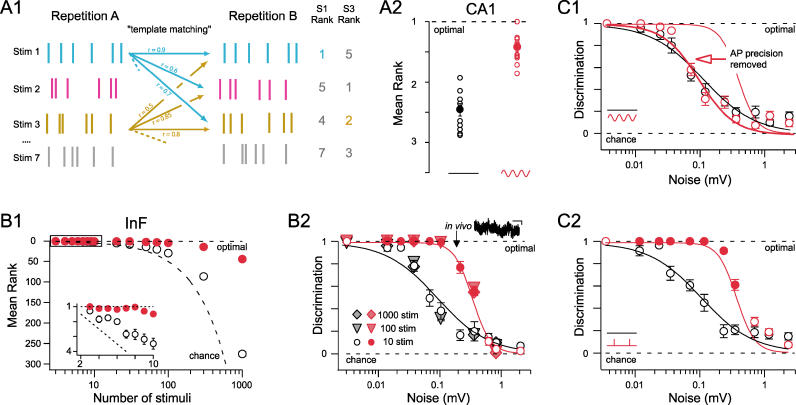
Oscillations Boost Stimulus Discrimination by Optimizing AP Precision (A1) Scheme showing the template-matching discrimination analysis: Spike trains of repetition A are correlated to trains from repetition B. Correlation coefficients are ranked and the rank of the correct match indicates level of discrimination: Examples shown are perfect (stimulus 1, highest correlation coefficient between 1A and 1B → rank 1) and near-perfect (stim. 3, second-highest correlation coefficient for 3A and 3B → rank 2). (A2) Stimulus discrimination plotted as the mean rank for the ten most separable stimulus sets from a single CA1 cell in vitro under control (no oscillation) and oscillation conditions ( *p* < 10 ^−12^ for all *n* = 135 stimulus sets in *n* = 3 cells). (B1) Stimulus discrimination plotted as mean rank as a function of stimulus number in simulation in an InF neuron. Inset: Mean rank scores for low numbers of stimuli. For all panels, filled red circles indicate where the discrimination for the oscillation case is significantly better than the respective control discrimination ( *p* < 0.05, Mann-Whitney). Dotted lines indicate perfect and chance levels of stimulus discrimination. (B2) Discrimination in an InF neuron is plotted against injected noise levels (Gaussian noise, low pass filtered at 830 Hz, value given is SD of membrane potential) for ten (circles), 100 (triangles), and 1,000 (diamonds) stimuli with (red) and without oscillatory current injection (black open circles, black/gray-filled triangles and diamonds). Solid lines are sigmoidal least square fits. The inset trace (top right) is an example of the in vivo membrane potential (recorded in the presence of TTX) used to estimate realistic noise levels (arrow). This level of noise is used in all simulations unless otherwise stated. Scale bar is 200 ms and 200 μV. Dotted lines indicate optimal and chance levels of stimulus discrimination. (C) Increased discrimination in InF neurons is due to enhanced AP precision. (C1) AP precision was measured for control and oscillation cases. The difference in AP precision was subtracted (jitter was added to the output spikes as described in Materials and Methods) from the oscillation case so that overall AP precision was identical to control conditions and the simulation was re-run. The sigmoidal fit to both oscillation data (red) and control data (open circles) is plotted. Sigmoidal fits from B2 are provided for comparison. (C2) In this simulation, the oscillatory drive was replaced by brief, large amplitude current injections every 250 ms to evoke precise APs. Discrimination is plotted as a function of membrane noise and is indistinguishable from the oscillation case [F _(1,120)_ = 1.32, *p* > 0.25]. Lines indicate best fits of the discrimination data for oscillation and nonoscillation conditions shown in B2.

Thus far, we have examined the separability of two different stimuli by comparing PSTHs compiled from many repetitions of each stimulus. Does this improved temporal separation of spike trains actually permit reliable readout of different stimulus situations in a single trial? To address this question we analyzed the in vitro recordings using a template-matching scheme, essentially asking; “Can one determine whether a spike train resulting from stimulus 1 was actually generated by stimulus 1 or by stimulus 2, 3, and so on?” Since our analysis requires discharge patterns generated in response to multiple stimuli, we restricted it to those cells in which responses to the most stimuli could be recorded. For each stimulus, two repetitions (A and B) were recorded using different random seeds for noise generation ( Figure 6A). The spike discharge pattern in response to stimulus 1 (noise A) was then cross-correlated with all discharge patterns for stimuli 1 to 7 (noise B). Reliable stimulus discrimination based on these correlations is possible only if the correlation between 1A and 1B is greater than all other correlations. Based on the number of stimuli that are more similar to stimulus X (noise A) than the repetition of stimulus X (i.e., noise B), we can attribute a “rank value” to the discrimination task. Rank values range from 1 (optimal match) to the number of stimuli ( *N*) compared; chance performance results in a mean rank of ( *N* + 1)/2. From this analysis of the in vitro data ( Figure 6A2) it is apparent that, in the absence of an oscillation, rank values are obtained that are close to those obtained by chance. In the presence of an MPO, many cases are observed in which stimulus discrimination is perfect or near-perfect (indicated by a mean rank score less than 2; Figure 6A2). Overall, stimulus discrimination as measured by the mean rank value is dramatically enhanced by the presence of an oscillatory drive ( *n* = 3 cells; 135 stimulus comparisons, *p* < 10 ^−14^).

For many neurons, the potential number of different input patterns to be discriminated will exceed the number of stimuli that can be experimentally presented during an in vitro recording. To determine whether oscillations actually enhance the coding capacity of cells, we took advantage of computer simulations that permit a detailed analysis of the importance of specific cellular and stimulus parameters. In a first step we used an integrate-and-fire (InF) model neuron as it is both general and computationally efficient. As with the in vitro experiments, Poisson-train stimuli were used to generate EPSP input under realistically noisy conditions (σ = 0.2 mV) to determine whether the postsynaptic neuron can readily discern different input stimuli based on its firing pattern. Again as in the experimental situation, sinusoidal current was injected into the soma to mimic the oscillation. Without an oscillation present, we found that it was impossible for the postsynaptic cell to discriminate the presented stimuli ( Figure 6B1, open circles). If, however, an MPO was introduced, we again observed almost perfect levels of stimulus discrimination ( Figure 6B1, red circles). Furthermore, the difference between the control and oscillation case increased drastically with larger numbers of stimuli [ Figure 6B1; F _(14,270)_ = 2,194, *p* < 10 ^−6^, two-way ANOVA]. Thus, these simulations in simple InF neurons not only reproduced the in vitro data but also show that by ensuring near-perfect discrimination between large numbers of stimuli, MPOs actually increase the coding capacity of a given neuron.

To obtain a direct measure of discrimination that is independent of stimulus space size we converted the mean rank *(r)* into a “discrimination score” *(d),* ranging from 0 (chance performance) to 1 (perfect discrimination; see Materials and Methods). We find that in the absence of any oscillatory drive, discrimination between stimuli deteriorates rapidly when realistic levels of noise are introduced [F _(14,126)_ = 50.7, *p* < 0.001, Figure 6B2]. Again, a background oscillatory current ensured almost perfect discrimination for all relevant noise levels [ Figure 6B2, F _(1,120)_ = 94.2, *p* < 10 ^−6^]. Discrimination values were up to ten times higher than in the absence of an oscillation and independent of the number of stimuli tested (compare circles, triangles, and diamonds in Figure 6B2). This allows for stimulus space-independent comparisons of stimulus discrimination in the absence and presence of the oscillation. Virtually identical results were obtained when we carried out the same analysis using five other types of published single- or multi-compartment model neurons ( Figure S1). When alternative discrimination assessment strategies including spike distance metrics [ 47] or mutual information [ 12, 48] were implemented ( Figure S2), similar results were achieved. Furthermore, as with AP precision ( Figure 1), using *oscillatory input trains* to generate background oscillations produced the same improvement in pattern discrimination as that achieved by “intrinsic” (injected) oscillations ( Figure S3).

### Stimulus Discrimination Relies on MPO-Mediated AP Precision

Having established that oscillations optimize AP precision, both in vivo and in vitro, it is tempting to propose that the enhanced discrimination is actually due to optimized AP precision. To confirm this we calculated AP jitter in the oscillation and nonoscillation cases in the simulated InF neuron. We found that for the oscillation case, AP precision was again enhanced (unpublished data). Preventing this increase in precision, by imposing additional AP jitter in the oscillation case, resulted in discrimination levels identical to the nonoscillation case [noise range 0.012 to 0.360 mV; F _(1,120)_ = 0.009, *p* > 0.9; Figure 6C1]. In the in vitro experiments, we found that replacing the oscillation cycle with precise APs (generated by very brief and large current injections) abolished jitter accumulation and recovered the precision of subsequent APs (see Figure 4D). This mirrored the precision enhancement with oscillatory current injections and synaptically driven oscillations. In simulations, we replaced the oscillatory drive with phase-locked APs whose timing was independent of the actual stimuli used. In agreement with the experimental data, these brief current pulses again recovered AP precision for subsequent APs. Furthermore, stimulus discrimination levels were numerically identical to those achieved by the oscillation [F _(1,120)_ = 1.32, *p* > 0.25, Figure 6C2]. Exactly the same result was obtained when the same number of large current injections were given at arbitrary times but fixed relative to the stimulus presentation (unpublished data). Together, these data indicate that MPOs permit stimulus discrimination *by increasing AP precision* and preventing jitter accumulation.

### The Dependence of Discrimination on MPO Parameters and Stimulus Locking

**Figure 7:**
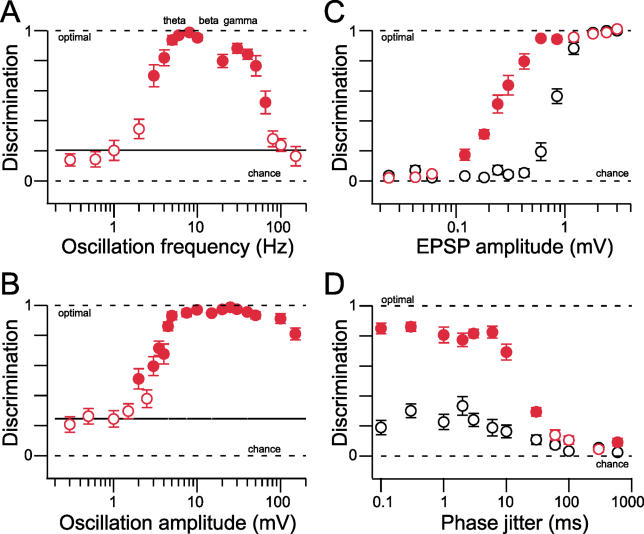
Improved Stimulus Encoding Is Largely Independent of Oscillation and Stimulus Properties (A) Stimulus discrimination in an InF neuron for ten different stimulus situations is shown as mean ± SEM for ten repetitions as a function of oscillation frequency. Horizontal black line indicates the mean discrimination level for the control case without oscillatory current injection. Filled red symbols indicate where discrimination for the oscillation case is significantly better than for the control case ( *p* < 0.05, Mann-Whitney). The terms theta, beta, and gamma indicate the respective physiologically relevant oscillation frequency ranges with respect to the levels of discrimination observed in the oscillation condition. (B) Same as (A) but with varying oscillation amplitude. (C) The dependence of stimulus discrimination on input strength in an InF neuron is shown by varying stimulus EPSP amplitude. For large inputs, discrimination is similar in the presence or absence of oscillatory activity, whereas the subthreshold drive significantly increases discrimination for lower EPSP amplitudes. (D) Influence of imperfect phase-locking of stimuli. Stimulus repetitions were presented after introducing a temporal shift (“jitter”) obtained from a Gaussian distribution with a width indicated as jitter (range: 0.1 to 600 ms). All other parameters, including noise, input and output firing rate, membrane time constant, EPSP kinetics, and amplitude, were kept constant (see Materials and Methods). Oscillation-enhanced discrimination begins to decline only when jitter values exceed 10 ms.

Making use of the flexibility simulations offer, we next asked what parameters of the oscillation were important for MPO-mediated discrimination. In a first step, we varied the oscillation frequency from 0.3 to 150 Hz while maintaining a peak-to-peak amplitude of 10 mV or changed oscillation amplitude, keeping frequency constant. In both cases, we observed near-perfect stimulus discrimination across most reported physiologically relevant frequencies (3 to 65 Hz; *p* < 10 ^−6^, Figure 7A) and amplitudes ( *p* < 10 ^−6^ for amplitudes greater than 1.5 mV, 4-Hz oscillation, Figure 7B). Varying the overall input Poisson train frequency revealed a range of overall spike rates that appears to be physiologically highly plausible (3 to 300 Hz, unpublished data). The resultant synaptic currents were also manipulated in different ways. First, we varied EPSC amplitude in the absence and presence of a constant 10-mV peak-to-peak oscillation. As expected, increasing EPSP amplitude (and thus increasing signal-to-noise) consistently improved discrimination in both the absence and the presence of a background oscillation ( Figure 7C). Interestingly, it was also across the physiologically most relevant ranges of unitary EPSP amplitudes that we observed a dramatic enhancement in stimulus discrimination in the presence of an oscillation ( Figure 7C), and varying the decay time of the EPSP failed to prevent this enhancement ( Figure S4). However, discrimination deteriorated for slower EPSPs, and in a more pronounced way for the nonoscillation case ( Figure S4). Together, these data show that under oscillatory conditions, enhanced stimulus discrimination occurs within physiologically realistic ranges of firing rates, EPSP kinetics, and oscillation parameters.

Thus far, we have used stimuli that were tightly locked to the ongoing oscillatory drive since input synchronization with a precision of less than a few milliseconds is frequently reported [ 49] and communication in many brain regions is thought to occur in “bursts” of activity tightly locked to an ongoing rhythm [ 50, 51]. However, to determine the importance of such locking for MPO-mediated discrimination, for stimulus repetitions we temporally shifted the input trains relative to the oscillation (i.e., we imposed a random “phase jitter” from 0.1 ms to 1 s). This resulted in no substantial alteration in the levels of MPO-mediated discrimination for jitter up to tens of milliseconds ( Figure 7D). It also indicates that MPO-mediated discrimination does not require unrealistic locking of stimulus input trains to the ongoing oscillatory rhythm.

### The Role of Oscillation Phase in Stimulus Discrimination

**Figure 8:**
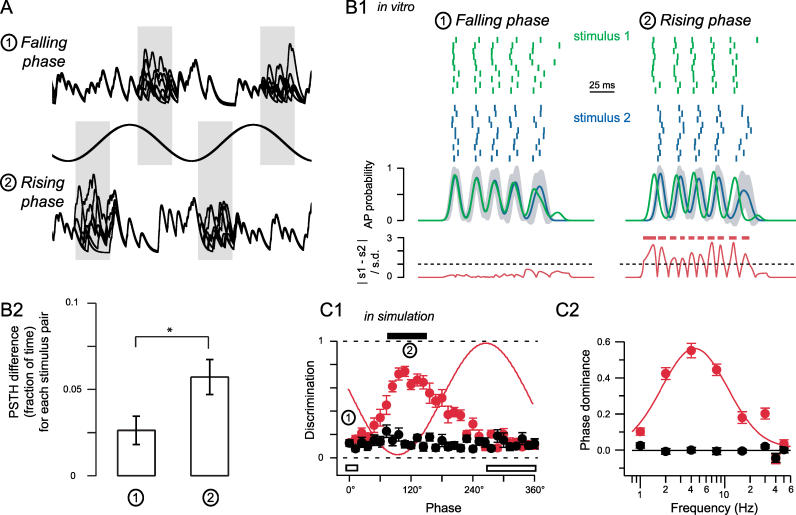
Oscillation Phase Is Critical for Optimal Discrimination (A) Two examples of input waveforms, each containing seven stimulus input trains (overlaid) that differed only during the indicated 100° windows (centered around −20° for the peak/falling phase situation (1) and 160° for the rising phase situation (2); the phase was measured relative to the downward midline crossing of the oscillatory drive). (B1) Rasters from an in vitro recording from a CA1 pyramidal neuron. The two stimuli only differ in the phase window indicated in (A). (Middle panels) PSTHs for both stimuli; the gray shading indicates the SD of the PSTH for stimulus 2. Note that while for the falling phase (1) both PSTHs overlap, they are easily separable for stimuli differing in the rising phase (2). (Bottom panels) Difference between the two PSTHs normalized to the combined SD. Red bars indicate where this normalized difference is larger than 1, that is, regions where the two stimuli are discernible based on the PSTHs. (B2) Summary of the in vitro recordings for the PSTH difference (the fraction of time where the normalized difference between the two PSTHs is larger than 1) for 22 stimulus pairs. The PSTH difference for rising phase stimuli is significantly larger than for falling phase stimuli ( *p* < 0.05). (C1) Stimulus discrimination is plotted under control and oscillation conditions as a function of the phase of the window for simulations in an InF neuron. A sinusoidal fit to the membrane potential is shown as a thin red line for reference. Stimulus windows similar to those shown in (A) are indicated by (1) and (2). To allow for higher temporal resolution the phase window was reduced to 60° and varied in steps of 12° across the cycle. Phase is measured relative to the downward midline crossing of the MPO. Solid bar indicates a period of best discrimination, while the open bar shows the period for the oscillatory case where discrimination is relatively poor. (C2) A sinusoid is fitted to the discrimination–phase plot. “Phase dominance” is defined as the peak-to-trough amplitude of the fitted sinusoid and plotted as a function of the oscillation frequency. As a control, the black line indicates the average phase dominance in the absence of a MPO. The red line is a Gaussian fit on a lin-log scale.

Having established that oscillations permit temporal separation of different stimulus situations, we then asked whether synaptic inputs arriving at different phases of an oscillation cycle were equally well discriminated ( Figure 8). In in vitro experiments, we therefore compared stimuli that differed solely during either the falling or rising phase of the oscillatory drive ( Figure 8A1 and 8A2, respectively). Stimuli that only differed in the falling phase showed strongly overlapping PSTHs, while stimuli differing in the rising phase of the oscillation resulted in clearly separable PSTHs (compare green and blue in Figure 8B1). Quantifying the PSTH difference we found it to be more than double for stimuli differing in the rising phase compared to the falling phase (2.6 ± 0.8% versus 5.7 ± 1.0%, *n* = 22 stimulus pairs; *p* < 0.05; Figure 8B2). To further assess the phase dependence of discrimination we used simulations with stimuli varying at different phases across the entire oscillation cycle ( Figure 8C1 and 8C2). In support of the in vitro data, we found that those EPSPs evoked in the trough and the early period of the rising phase were most reliably discriminated, while those inputs arriving at the peak and the early falling phase of the cycle were the least discriminated (0° versus 120°, *p* < 10 ^−7^, Figure 8C1). In a final steps, we varied the underlying oscillation frequency. For low oscillation frequencies, we find that the relative phase of synaptic inputs strongly determines the extent to which they will be discriminated ( Figure 8C2). It was only at frequencies above 20 Hz that this phase-relation began to “smear out” over the entire cycle ( Figure 8C2). For all physiological oscillation frequencies, however, discrimination across the entire oscillation cycle was never worse than for the control case. These data indicate that particularly for low oscillation frequencies, the timing of inputs relative to the phase of an oscillation cycle may play an important role in determining the accuracy of stimulus discrimination.

## Discussion

Neuronal oscillatory activity is considered a physiological hallmark of sensory, motor, and cognitive function [ 31, 32, 35, 39, 52– 60]. Here we have shown that oscillations ensure optimal levels of AP precision that in turn permit very high levels of stimulus discrimination. This was independent of whether oscillations were directly injected in vitro or whether the actual input stimuli provided the oscillatory drive. Mechanistically, oscillations achieve this as they establish a period of hyperpolarization that prevents the accumulation of jitter otherwise inherently associated with ongoing spike trains.

We found that MPOs enhance discrimination in real neurons and in simulation using InF or conductance-based models ( Figure S1). Such discrimination relies on an increase in AP precision ensured by the ongoing MPOs. In InF neurons, every AP is associated with a complete reset of the membrane potential. Imprecision in AP time produces variability in the onset of the next integration window. Therefore, the time of a given AP will be not only variable due to ongoing background noise but also related to the variability of the previous spike. Thus, in both real [ 10, 13] and InF neurons, jitter accumulates across ongoing spike trains. Here we have examined how MPOs might prevent such jitter accumulation. By studying the time course of this interaction, we find that the minimum hyperpolarization time necessary to fully recover AP precision is approximately 20 ms. This reflects the time needed to restore coherence in the relevant membrane properties, such as to ensure full decay of voltage variability ( Figure 4A) or to allow, for example, Na ^+^ channels to recover from inactivation associated with an AP.

The specific role of oscillations has long been debated. Such rhythmic activity ranges from theta (1 to 10 Hz) [ 37– 39, 42, 52, 53, 61] to gamma frequencies (>40 Hz) [ 31, 32, 54, 62, 63] and has been observed in single cells, both in vivo and in vitro *,* and across neuronal networks [ 64– 69]. At the network level, various roles for such activity have been suggested and most notably include the synchronization of stimulus relevant patterns of activity [ 31, 32, 56, 70]. In this scheme oscillatory rhythms are used by neuronal networks to temporally bind together the firing of a specific subset of cells that are activated under a specific set of stimulus-driven conditions. At the single-cell level, oscillatory activity has been described as rhythmic almost sinusoidal deflections of the membrane potential, generated both synaptically [ 37, 42, 66, 71– 73] and by intrinsic mechanisms [ 57, 58, 74– 76]. In the hippocampus and the olfactory system, for example, such oscillations at the individual cell level are suggested to serve as an internal reference signal whereby spike trains could encode information by their phase relative to the background oscillation cycle [ 31, 35, 37, 39]. In the hippocampus, this type of phase coding is believed to underlie the representation of an animal's location in space [ 38, 39]. Here, a temporal code might be generated from a firing rate code by means of an inhibitory oscillatory drive [ 38]. In the olfactory bulb, phase coding could be used to reflect the relative amount of sensory input to a mitral cell [ 37, 42]. Therefore, the timing of synaptic input—relative to an oscillation cycle—might be a useful means of representing stimulus-specific information [ 3, 35, 60, 77, 78]. The oscillation-mediated enhancement of precision described here will contribute to enhancing the fidelity of such phase codes by increasing the precision of the phase relative to the theta rhythm.

The interplay between excitatory and inhibitory connections and an intrinsic resonance might be sufficient to establish a network of synchronously oscillating neurons [ 66, 79– 81]. Individual interneurons have been shown to efficiently coordinate activity by providing postinhibitory rebound activation in pyramidal neurons in hippocampus [ 82]. As these pyramids will subsequently activate interneurons, the increase in precision of AP discharge might facilitate the synchronization of oscillations across such networks.

AP precision is often thought to be the limiting factor in maximizing neuronal coding capacity [ 5, 83]. Various mechanisms for attaining high AP precision have been suggested. These include synchronization of discharge via electrical gap junctions [ 79, 80, 84, 85] and feed-forward and recurrent inhibition [ 86, 87]. Both mechanisms may further enhance the robustness of the oscillation and/or directly improve AP precision. The maintenance of high levels of AP precision will also allow for easy downstream readout through, for example, a delay-based mechanism relying on synchrony detection [ 33, 35, 37] or when a postsynaptic integrator is explicitly provided with the phase of the oscillatory drive from a common rhythm generator. For slow oscillations both in vivo and in vitro, the first spike in every cycle was more precise than later spikes. Although progressively less precise, such late spikes may still provide substantial information about the stimulus; readout mechanisms might also benefit from the occurrence of multiple spikes through, for example, a depolarizing trigger for short- and long-term plasticity [ 88, 89].

In mitral, CA1 pyramidal, and Purkinje cells, we found that oscillations ensure optimal levels of AP precision during ongoing spike trains. The membrane potential variance immediately preceding a depolarizing cycle was found to be the key factor in determining the degree of precision of the next AP. Since the recovery of membrane potential variance was highly correlated with the time course of the AHP, it is likely that the time course of precision recovery will vary depending on the intrinsic conductances in a given cell type. For example, in mitral cells, recent work has shown that an intricate interplay of slow I _d_-like K ^+^ and subthreshold Na ^+^ currents shapes AP clustering and precision and tunes mitral cells to respond most reliably to phasic stimuli [ 44]. Since we have shown that oscillation-mediated AP precision exists in very different cell types, both experimentally and theoretically, our observations may generalize to a range of neuronal populations with differing intrinsic properties.

In our experiments, oscillations were found to recover AP precision from an otherwise jittery spike train, with a minimum recovery time period of approximately 20 ms. This implies that the maintenance of AP precision could be compromised at oscillation frequencies higher than about 50 Hz. Our finding that stimulus discrimination starts to deteriorate at oscillation frequencies above 65 Hz supports this. One must also expect that the time constant of precision recovery depends to some extent on the membrane time constant. In simulations, varying the membrane time constant across a physiologically relevant range (8 to 80 ms) was found to have no effect on the enhanced level of discrimination ensured by oscillations (unpublished data). Again, it seems likely that the membrane time constant will significantly impact oscillation-mediated AP precision only at high oscillation frequencies.

From recordings in vitro and in simulations, oscillations unambiguously and dramatically enhanced discrimination between subtly different input trains by maintaining optimal levels of AP precision. This was independent of whether we compared differences in PSTHs or used a template-matching scheme, spike-distance metrics, or information theory approaches. Since smoothing the spike trains with sliding windows of up to 100 ms did not qualitatively alter this finding, it appears that oscillations are beneficial for discrimination not only for “AP timing codes” but also for “rate codes” that read out average firing rate across time windows of 10 to 100 ms.

For theta oscillation frequencies, it was those inputs arriving during the trough and rising phase that were best discriminated. This is likely to be due to the fact that input arriving on the early falling phase and the very beginning of the hyperpolarizing trough will have largely decayed and thus not significantly contribute to the discharge of the next AP (the first AP of the succeeding cycle). Inputs in the trough or rising phase arrive during the core of the integration window and thus strongly impact the APs. This was particularly pronounced for lower frequencies, indicating that the time window for integration is rather large and provides an opportunity for many inputs to contribute to the resultant output. Thus, it seems that for theta frequencies in particular, the phase of both inputs and outputs [ 35, 36, 38, 90] are relevant for encoding information. These results held true not only for EPSPs but also if purely inhibitory or a mixture of excitatory and inhibitory input was used (unpublished data). Furthermore, there was no measurable difference in the overall discrimination levels between EPSP- and inhibitory postsynaptic potential–based stimuli (not shown). This indicates that under oscillatory conditions, neurons can distinguish equally well the subtle temporal features of excitatory and inhibitory input. In the case of EPSPs, it seems likely that if individual events are large enough or if only a small number of EPSPs are needed to reliably exceed threshold [ 13, 23], a different phase relation might be found. Our data show that, despite its phase and oscillation frequency, input discrimination of both EPSPs and inhibitory postsynaptic potentials is never worse than in the nonoscillation case, the only potential exception being very long oscillation periods (<2 Hz). This also indicates that, although providing a “window of opportunity” for integration, the presence of an oscillatory drive generally does not create “information holes” as one might have expected.

Based on our experiments involving the manipulation of the EPSP time course, it seems that slow NMDA-like inputs are less well discriminated than α-amino-3-hydroxy-5-methyl-4-isoxazolepropionate (AMPA)-like EPSPs. Thus while the AMPA component might carry most of the stimulus-specific information that is present during signal processing, the NMDA component, via its Ca ^2+^ permeability, may be better suited to providing a trigger that signals a bidirectional change in synaptic efficacy [ 91– 94]. In this context, it is interesting that oscillations not only provide a time base for signaling the most readily discriminated input but also would ensure that the “precise APs” necessary for the induction of spike-timing–dependent plasticity (STDP) [ 95– 100] are evoked. In fact, the phase relation observed for optimal discrimination—EPSPs preceding the AP being the most influential—is reminiscent of the known requirements of STDP that are thought to play important roles in balancing overall levels of synaptic activity and the formation of sensory representations. Thus, STDP could be used to tune a given cell to stimulus-specific situations and ongoing oscillations might provide a physiologically plausible means of pairing input and APs over the many trials necessary to produce measurable changes in synaptic efficacy [ 95– 100].

The robustness and generality of our finding that oscillations enhance stimulus discrimination are probably due to the simplicity of the underlying mechanism. Oscillations achieve enhanced discrimination by providing periods of nonspiking that prevent the accumulation of AP jitter. This simple principle is likely to occur in any nonlinear system where information must be integrated and transformed into discrete output signals. Examples of such systems where fidelity benefits from intermittent resetting pauses and/or rhythmic cycles include computer clocks and rhythmic gene expression systems. From an information transmission standpoint, essentially any form of communication with intermittent “pauses” (e.g., Morse code) guarantees that errors are not accumulated and ensures reliability. In neurons, oscillations provide this intermittent pause of firing, reset precision and permit the accurate transmission of neural information.

## Materials and Methods

### In vivo electrophysiology.

Male and female C57Bl6 mice aged 3 to 5 wk were anesthetized using a ketamine (50 mg/kg)–xylazine (5 mg/kg) mixture and supplemented throughout the experiment. In both freely breathing and artificially respirated animals, a piezoelectric strap (WPI, Sarasota, Florida, United States) was placed around the thorax and was used to provide a respiration distention signal for recording the ongoing oscillation cycle. In freely breathing animals, odors were presented as previously described [ 37, 101]. In artificially respirated animals, the trachea was incised and cannulated with one tube directed to the lung and the other toward the pharynx, allowing for the precise control of odorized nasal airflow [ 40]. During whole-cell recordings, the online thorax signal was used to trigger the pulsed nasal inspirations at the natural frequency (oscillation case) or the odorized nasal airflow was kept constant with the same average flow rate (no oscillation case). In both cases, AP times were quantified using the recorded thorax signal as reference. The distribution of AP times around the mean AP time was used to measure AP precision: The mean distance reflects AP precision (low values indicating high precision) and its standard deviation or standard error is shown as error bars ( Figure 1D and 1E).

Whole-cell recordings were carried out as previously described [ 102] using low resistance pipettes (5 to 7 MΩ) containing (in mM) K-gluconate 135, HEPES 10, phosphocreatine 10, KCl 4, Mg-ATP 4, GTP 0.3 (pH 7.2). Recordings were amplified using an Axoclamp 2B amplifier (Axon Instruments, Union City, California, United States) in bridge mode, filtered at 3 to 10 kHz, and digitized at 5 to 20 kHz (ITC-16; InstruTECH, Port Washington, New York, United States). Data were acquired using Heka software (Heka Elektronik, Lambrecht, Germany) running on a Macintosh computer. In both the oscillation and nonoscillation case, constant current was sometimes injected to drive cells to threshold so APs could be recorded and the number evoked per respiration cycle could be controlled. Precision of AP timing was thus measured across cycles with the same number of APs. To estimate realistic levels of membrane noise, membrane voltage was recorded in the absence of AP-dependent synaptic activity after superfusion with 20 μM TTX applied to a bath over the olfactory bulb [ 101].

### In vitro electrophysiology.

In vitro recordings were carried out in horizontal olfactory bulb, transverse hippocampal, and sagittal cerebellar slices from C57Bl6 mice aged P18 to P30. Slices (300 μm thick) were cut and incubated for 45 min at 35 °C in standard external recording solution (125 mM NaCl, 2.5 mM KCl, 2 mM CaCl _2_, 1 mM MgCl _2_, 25 mM NaHCO _3_, 1.25 mM Na _2_PO _4_ and 25 mM d-glucose) as described previously [ 101]. During recording, slices were continuously perfused with external solution heated to 32 ± 1 °C, bubbled with 5% CO _2_ and 95% O _2_. Patch pipettes had tip resistances of 4 to 8 MΩ when filled with internal solution containing (in mM) K methansulfonate 130, HEPES 10, KCl 7, EGTA 0.05, Na _2_-ATP 2, Mg-ATP 2, GTP 0.5, and Biocytin 0.4% (pH 7.2 with KOH). Voltage recordings were obtained from cell somata in the whole-cell configuration using a Multiclamp 700A amplifier (Axon Instruments). The signal was filtered at 3 to 10 kHz and digitized at 5 to 20 kHz (ITC-18; InstruTECH). Measured potentials are not corrected for junction potentials. Data were acquired using Axograph (Axon Instruments) software or custom software in Igor (WaveMetrics, Lake Oswego, Oregon, United States) running on a Macintosh computer. To estimate AP jitter in vitro, we used the minimal point of the trough of the injected current in the oscillation case as the beginning of the oscillation cycle ( Figure 2). These same reference time points were used in the control case and AP precision was measured as in the in vivo situation. In both cases precision was normalized to that expected by chance. Subsequently, AP precision for the first–fourth AP per cycle was combined for cycles containing one to five APs and normalized to the average control case. As an alternative measure (e.g., Figure 5B), precision was measured as previously described [ 13] with event thresholds adjusted to result in 90% reliability. Recovery of precision in Figure 3 was measured as (jitter of third − fourth)/(third − first AP).

### Stimulus generation.

Stimulus spike trains were drawn from Poisson distributions with a total mean firing rate of 100 Hz. Input currents were generated by convoluting these spike trains with an EPSP-like waveform [v _EPSP_(t) = (1 − *e* ^−t/10 ms^) · *e* ^−t/8 ms^]. Gaussian noise filtered at 830 Hz with a root-mean-square of typically 20 pA was added to the resulting wave. For the oscillation case, a 4-Hz oscillation was added with the amplitude adjusted to yield an approximately 10-mV peak-to peak amplitude. Finally, DC levels were manually adjusted to yield an output firing rate that was constant (±3%) for repetitions, for different stimuli, and for the oscillation and control case in a given cell.

### Mean rank.

Spike trains were determined for seven stimulus situations, each repeated once with a different random seed to generate the noise, resulting in spike trains 1A, 1B, 2A, 2B, … 7A, 7B. Spike train 1A was then correlated with the templates 1B, 2B, …7B resulting in seven correlation coefficients *r _1(1)_, r _1(2)_, r _1(3)_* … *r _1(7)_*. High correlation coefficients indicate high similarity and will be maximal for *r _1(1)_* in the absence of corrupting noise. Therefore, the degree of noise corruption can be expressed in the rank of *r _1(1)_*, calculated by sorting the correlation coefficients in descending order and measuring the position of *r _1(1)_* in that order. If noise levels are high, this will result, on average, in a rank of 4. Lower ranks point to a similarity between spike train 1A and 1B that is higher than expected by chance. Rank values of 1 indicate that spike train 1A is more similar to template 1B than to any other template AP pattern. This procedure is repeated by comparing spike train 2A to templates 1B, 2B … and similarly for spike trains 3A… 7A, resulting in seven rank values. The mean rank value for the seven stimuli therefore ranges from 1 to 7, a rank of r _chance_ = 4 reflecting chance discriminations. If more than two repetitions were acquired this process was repeated for repetitions C and D … etc. Spike trains were correlated for comparison after capture with a sliding window. Although rather insensitive against changes in filter, window width ( Figure S2B) usually mean ranks for 2, 5, and 50 ms were calculated for the oscillatory and the nonoscillatory case and the window size resulting in the lowest rank was used.

### PSTH difference.

Spike trains were determined for ten repetitions for two stimuli and convoluted with a Gaussian (σ = 5 ms). The ten repetitions were averaged resulting for each stimulus in a mean PSTH (PSTH1, PSTH2) and a respective standard deviation (SD1, SD2). To compare the two stimuli, the weighed difference d = (PSTH1 − PSTH2)/SD was calculated for each time point (SD ^2^ = SD _1_^2^ + SD _2_^2^). The fraction of time during which this weighed difference exceeds 1 is a measure for the discriminability of the two stimuli and was calculated for all possible stimulus pairs, e.g., (7 × 6)/2 = 21 for seven stimuli.

### Simulations.

Simulations were carried out using Matlab 6.5 (The MathWorks, Natick, Massachusetts, United States) in leaky InF neuron models using the csim_lifnet simulation tool (T. Natschläger, available at http://www.igi.tugraz.at/tnatschl) with *τ _membrane_* = 30 ms, *V _threshold_* = 15 mV, *V _reset_* = 5 mV, and a refractory period of 5 ms. Stimuli were generated as in the in vitro experiments with a synaptic time constant of *τ _Synapse_* = 10 ms. Results from more complex cellular models are described in detail in Supporting Information. To measure precision in the simulations ( Figure 6C1), precision of the first, second, third, and fourth APs was determined and averaged. To subtract the precision difference, a normally distributed time was added to output spike times. The width of this distribution was determined by calculating the variance in overall AP discharge times, which we refer to as “jitter.” Jitter between the control case and the oscillation was normalized for the uneven distribution of spikes (85% of spikes fall during 85% of the cycle time for the control case but are condensed to only 63% of cycle time in the oscillation case; see, e.g., Figure 1C). Use of alternative measures such as reliability and precision [ 13] or variance versus mean count plots [ 12] perfectly reproduced the oscillation-mediated enhancement of AP precision. Mean ranks were determined as in the in vitro experiments but, unless otherwise stated, ten simulation runs with different random seeds were averaged for ten stimuli each. Discrimination *(d)* was calculated from the rank value *(r)* where *d* = ± [1 − ( *r* −1)/( *r _chance_* − 1)] ^2^ and the sign was determined by whether *r* was larger (+) or smaller (−) than chance levels.

## Supporting Information

Figure S1 Oscillations Enhance Discrimination Irrespective of Cell Type or Intrinsic Cellular Properties (A) Morphologies of neuron models used. Scale bar = 100 μm. (B) Firing pattern in response to threshold depolarizing and hyperpolarizing step current injection (from left to right: ±15 pA, ±380 pA, −100/+200 pA, ±100 pA, +200/−100 pA, ±400 pA). (C) Resonance properties. (D) Discrimination as a function of noise. InF neuron is the same as in Figure 6B2 and is displayed for comparison.
(419 KB PDF)

Figure S2 Oscillations Enhance Discrimination Independent of Assessment Method (A1) The PSTH difference in an InF neuron for seven stimuli (21 stimulus pairs) is plotted against the simulated noise (Gaussian noise, low pass filtered at 830 Hz, value given is SD of subthreshold membrane potential). Shown is mean ± SEM for ten repetitions at each noise level with (red) and without (black open circles) oscillatory current injection. For all panels, filled red symbols indicate where the discrimination for the oscillation case is significantly better than the respective control discrimination ( *p* < 0.05, Mann-Whitney). The arrows indicate noise levels estimated from in vivo recordings. (A2) Mutual information between spike trains and stimuli [ 12] was calculated as a function of background noise levels. The presence of the subthreshold drive results in an increase of mutual information consistent with the increased stimulus discrimination (A4). (A3) (Left) One hundred stimuli were presented and distances between the resulting spike trains were calculated with (red open circles) and without (black open circles) oscillatory current injection [ 47]. Similarly, one stimulus was repeated 100 times in the presence of noise (0.12 mV, solid circles) and distances between the resulting spike trains were calculated. Note that distances are virtually unchanged ( *p* = 0.7) in the absence of oscillations, indicating that different stimuli cannot be discriminated. Oscillations improve discrimination as measured by the decreased distance between spike trains resulting from repetitions ( *p* < 10 ^−5^). In the bar chart on the right, mean ± SEM of the differences between “different stimuli” and “repetitions” is depicted. (B) Influence of oscillations on stimulus discrimination as a function of the filter width used for spike train comparison.
(279 KB PDF)

Figure S3 Synaptic Oscillations Can Effectively Substitute for Sinusoidal Current Injection (A1) Presynaptic firing for one stimulus consisting of excitatory and inhibitory inputs. Firing rates are drawn from Poisson distributions with a modulated firing rate proportional to (1 + *S* · sin ^3^(±2πft) with *S* being the strength of the modulation) for excitatory and inhibitory inputs, respectively. *S* was (from top to bottom) 0, 0.3, and 1. Total mean firing rate was in all cases maintained at 100 Hz. (A2) Input currents evoked by the three stimulus situations shown in A1. EPSCs are displayed as upward deflections. (A3) Resulting membrane potential corresponding to the three conditions. Hyperpolarizing current was injected to extract the subthreshold membrane response. (B) Discrimination values for the three situations depicted in (A) and ten different stimuli. (C) Same as in (B) for the strong modulation case with varying noise levels. Note that the noise dependence of the discrimination value is identical to that observed with direct sinusoidal current injection ( Figure 6B2).
(333 KB PDF)

Figure S4 Oscillations Improve Discrimination for Fast and Slow EPSPs Discrimination was measured for an InF neuron as a function of the time constant of the EPSP.
(78 KB PDF)

Protocol S1 Supporting Results and Methods Analysis showing that stimulus discrimination is improved by MPOs irrespective of cell types, assessment methods, and origin of oscillations.
(95 KB PDF)

## Abbreviations

AHP: afterhyperpolarization
AMPA: α-amino-3-hydroxy-5-methyl-4-isoxazolepropionate
AP: action potential
EPSP: excitatory postsynaptic potential
InF: integrate-and-fire
MPO: membrane potential oscillation
NMDA: *N*-methyl-d-aspartate
PSTH: peristimulus time histogram
STDP: spike-timing–dependent plasticity
Vm _post_: posthyperpolarization membrane potential
Vm _pre_: prehyperpolarization membrane potential
