# Supplementary material for: Neuronal Oscillations Enhance Stimulus Discrimination by Ensuring Action Potential Precision
Source: PLoS Biol. 2006 May 16;4(6):e163. doi: 10.1371/journal.pbio.0040163 (PMC1459879; doi:10.1371/journal.pbio.0040163)
Supplement: Supplementary file 1 [file pbio.0040163.sd001.pdf]

Supporting Results and Methods

MPOs improve stimulus discrimination irrespective of cell type

To assess whether the described enhancement of discrimination is dependent on intrinsic neuronal properties, we performed further simulations in a wide variety of cellular models (Figure S1) from simple single compartment integrate-and-fire neurons and isopotential cells containing channels following Hodgkin-Huxley kinetics, to complex compartmental models of mitral cells (Shen et al., 1999), dentate gyrus basket, hippocampal granule cells (Santhakumar et al., 2005), and CA1 pyramidal neurons (Traub et al., 2003). Note that these cell types have both very different morphologies (Figure S1A) and intrinsic parameters resulting in highly diverse firing properties, IV relations (Figure S1B) and intrinsic resonance properties (Figure S1C). As no qualitative difference in discrimination could be observed between the cell types tested (Figure S1D) the observed oscillation-mediated enhancement of discrimination is a general phenomenon independent of specific cellular properties. The heterogeneous resonance properties (Figure S1C) further suggest that there was very little interaction between injected oscillatory drive and intrinsic oscillations. This was confirmed in simulations where a combination of two oscillations with different frequencies was used. Discrimination was improved as predicted from the individual frequencies (Figure 7A, B and not shown).

MPOs enhance stimulus discrimination irrespective of assessment method

Using a template-matching scheme and an integrate-and-fire neuron we demonstrated that oscillations substantially enhanced discrimination for a wide range of oscillation and cellular parameters. To further assess whether discrimination was dependent on the choice of analysis method we determined the PSTH difference for simulated data from an integrate-and-fire neuron for the same noise range (Figure S2A1). The PSTH difference was substantially increased for the same noise range where Mean Rank based discrimination was improved by the oscillation (Figure 6). In addition, using information theory measures such as mutual information between stimuli and spike trains (Figure S2A2) showed that for the same noise range mutual information was enhanced in the presence of an oscillation. Another alternative comparison measure such as a spike distance metric (Victor and Purpura, 1997) was also used. Again, in the presence of an oscillatory drive distances between spike trains belonging to repetitions of the same stimulus were significantly smaller than distances between different stimuli (Figure S2A3, red, $p < 10^{-5}$). In the absence of an oscillation, distances between repetitions were indistinguishable from distances between spike trains belonging to different stimulus situations (Figure S2A3, black, $p = 0.7$). The distance difference (d.d.) between dissimilar stimuli and repetitions can be taken as an alternative measure of discrimination. Whereas in the absence of an oscillation no discrimination was possible (d.d. = 0.015 ± 0.05, mean ± sem, Figure S2A3, right), it was vastly improved in the oscillation case (d.d. = 2.14 ± 0.03, $p < 10^{-202}$). For direct comparison with the discrimination scheme used in the core manuscript we reproduced Figure 6B2 for 10 stimuli (Figure S2A4).

To compare spike trains for our standard discrimination measurement (Figure 6A1), rasters were binned and correlated. Figure S2B shows that if the binning width is altered from a few ms up to 100 ms, although essentially changing the neuronal properties from coincidence detection to integration (Gerstner et al., 1997; Konig et al., 1996; Rudolph and Destexhe, 2003), oscillations consistently provided superior computation. Thus, both for AP timing codes relying on information being contained in AP times with a necessary accuracy of few milliseconds and for firing rate codes relying on the AP discharge averaged over up to 100 ms, an oscillatory drive provides substantially improved discrimination.

Oscillatory modulation of the input trains enhances stimulus discrimination

We have shown *in vivo*, *in vitro* and in simulation that oscillations enhance AP precision. When oscillatory current was injected and stimulus waveforms tightly controlled, this increased AP precision dramatically enhanced discriminability. We also tested whether the input stimuli themselves could provide the oscillatory drive necessary for improved discrimination. Inputs consisted of inhibitory and excitatory inputs, both driven by Poisson spike trains with a sinusoidally modulated firing rate and an average firing rate of 100 Hz (Figure S3A1, A2). Varying the strength of the modulation resulted in improved discrimination when the levels of membrane potential oscillations reached those obtained from direct current injection ('strong modulation', Figure SA3, B). Similarly, if oscillation amplitudes reached those observed *in vivo*, discrimination was enhanced for all relevant noise levels (Figure S3C). Thus, both injected and synaptic oscillations enhance stimulus discrimination.

To further assess the importance of the EPSP time course we show that fast EPSPs (resulting in more strongly patterned input current) provide better discrimination than EPSPs with a slower kinetic (Figure S4). The presence of an oscillation, however, consistently improved discrimination irrespective of the time course.

Methods

Simulation parameters

All compartmental simulations were carried out in NEURON 5 (Hines and Carnevale, 1997) using a mitral cell (Shen et al., 1999), dentate gyrus basket and granule cell (Santhakumar et al., 2005) and CA1 pyramidal neuron model (Traub et al., 2003). For the single compartment models cellular parameters were, if not noted otherwise, as described in the main manuscript. A previously described Hodgkin Huxley model (provided by Michele Giugliano and obtainable through http://www.mathworks.com) was incorporated in the csim_lifnet environment and used in Figure S1. Stimulus strength and amplitude of the sinusoidal current injection were adjusted to yield the same membrane potential values as in the integrate-and-fire model. Resonance properties were measured by injecting sinusoidal currents of varying frequency with a peak-to-peak amplitude of <10 mV adjusted with DC current injection to result in subthreshold activity with peak depolarizations close (< 5 mV) to threshold. The complete Matlab and Neuron code can be obtained from the authors.

Measures to assess stimulus discrimination

Mutual information

Mutual information was calculated by standard means (de Ruyter Van Steveninck et al., 1997) from 100 different stimuli (total information) and 100 repetitions of one stimulus (noise entropy). Spike trains were binned with 1 ms bins, word sizes were 2, 3, 4, 5, 6, 7, 8, 9, 10, 15, 20 and 30. Both information and noise entropy were calculated by extrapolating the respective entropies for the given word sizes to infinite word size (de Ruyter Van Steveninck et al., 1997). Control simulations with 1000 stimuli / repetitions were performed to ensure reliable estimation of the entropies and yielded exactly the same result.

Spike distance metrics

To directly measure the distance between spike trains a standard metric was used (Victor and Purpura, 1997). In brief, two spike trains are compared by converting one spike train into the other following two rules: Shifting a spike by *n* ms is assigned a "cost" of $q*n$, Deleting or recreating a spike "costs" 1. In Figure 6A2 this punishment *q* was 0.1 so that a shift of 20 ms is as "expensive" as deleting and recreating a spike. The total cost to convert one spike train into another is referred to as the "distance" between the trains (Victor and Purpura, 1997). Distances were calculated for train pairs within a set of 100 *different* stimuli (open circles in Figure S2A3) or for spike train pairs within a set of 100 *repetitions* of one stimulus (' "within" repetitions of stimulus 1', filled circles). Discriminability of the 100 stimuli is guaranteed if and only if the distance between spike trains resulting from different stimuli is larger than the distance between spike trains that result from repeating the same stimulus. In this situation, given a spike train for an unknown stimulus, its distance to "template" spike trains could be calculated and the template that yields the lowest distance would be identified as the underlying stimulus. Since both spike distance metric and the mutual information measure have much higher computational requirements, the majority of results reported are based on the "Discrimination" template-matching scheme described in the main manuscript.

The mean rank and Discrimination measures as well as the PSTH difference determination are described in the main manuscript and used the same way for the simulated data as for the *in vitro* recordings.

References

de Ruyter Van Steveninck,R.R., Lewen,G.D., Strong,S.P., Koberle,R., and Bialek,W. (1997). Reproducibility and variability in neural spike trains. Science *275*, 1805-1808.

Gerstner,W., Kreiter,A.K., Markram,H., and Herz,A.V. (1997). Neural codes: firing rates and beyond. Proceedings of the National Academy of Sciences of the United States of America *94*, 12740-12741.

Hines,M.L. and Carnevale,N.T. (1997). The NEURON simulation environment. Neural computation *9*, 1179-1209.

Konig,P., Engel,A.K., and Singer,W. (1996). Integrator or coincidence detector? The role of the cortical neuron revisited. Trends in neurosciences *19*, 130-137.

Rudolph,M. and Destexhe,A. (2003). Tuning neocortical pyramidal neurons between integrators and coincidence detectors. Journal of computational neuroscience *14*, 239-251.

Santhakumar,V., Aradi,I., and Soltesz,I. (2005). Role of mossy fiber sprouting and mossy cell loss in hyperexcitability: a network model of the dentate gyrus incorporating cell types and axonal topography. Journal of neurophysiology *93*, 437-453.

Shen,G.Y., Chen,W.R., Midtgaard,J., Shepherd,G.M., and Hines,M.L. (1999). Computational analysis of action potential initiation in mitral cell soma and dendrites based on dual patch recordings. Journal of neurophysiology *82*, 3006-3020.

Traub,R.D., Buhl,E.H., Gloveli,T., and Whittington,M.A. (2003). Fast rhythmic bursting can be induced in layer 2/3 cortical neurons by enhancing persistent Na+ conductance or by blocking BK channels. Journal of neurophysiology *89*, 909-921.

Victor,J.D. and Purpura,K.P. (1997). Metric-space analysis of spike trains: Theory, algorithms and application. Network *8*, 127-164.
