# Supplementary material for: Neuronal Oscillations Enhance Stimulus Discrimination by Ensuring Action Potential Precision
Source: PLoS Biol. 2006 May 16;4(6):e163. doi: 10.1371/journal.pbio.0040163 (PMC1459879; doi:10.1371/journal.pbio.0040163)
Supplement: Supplementary file 2 [file pbio.0040163.sg001.pdf]

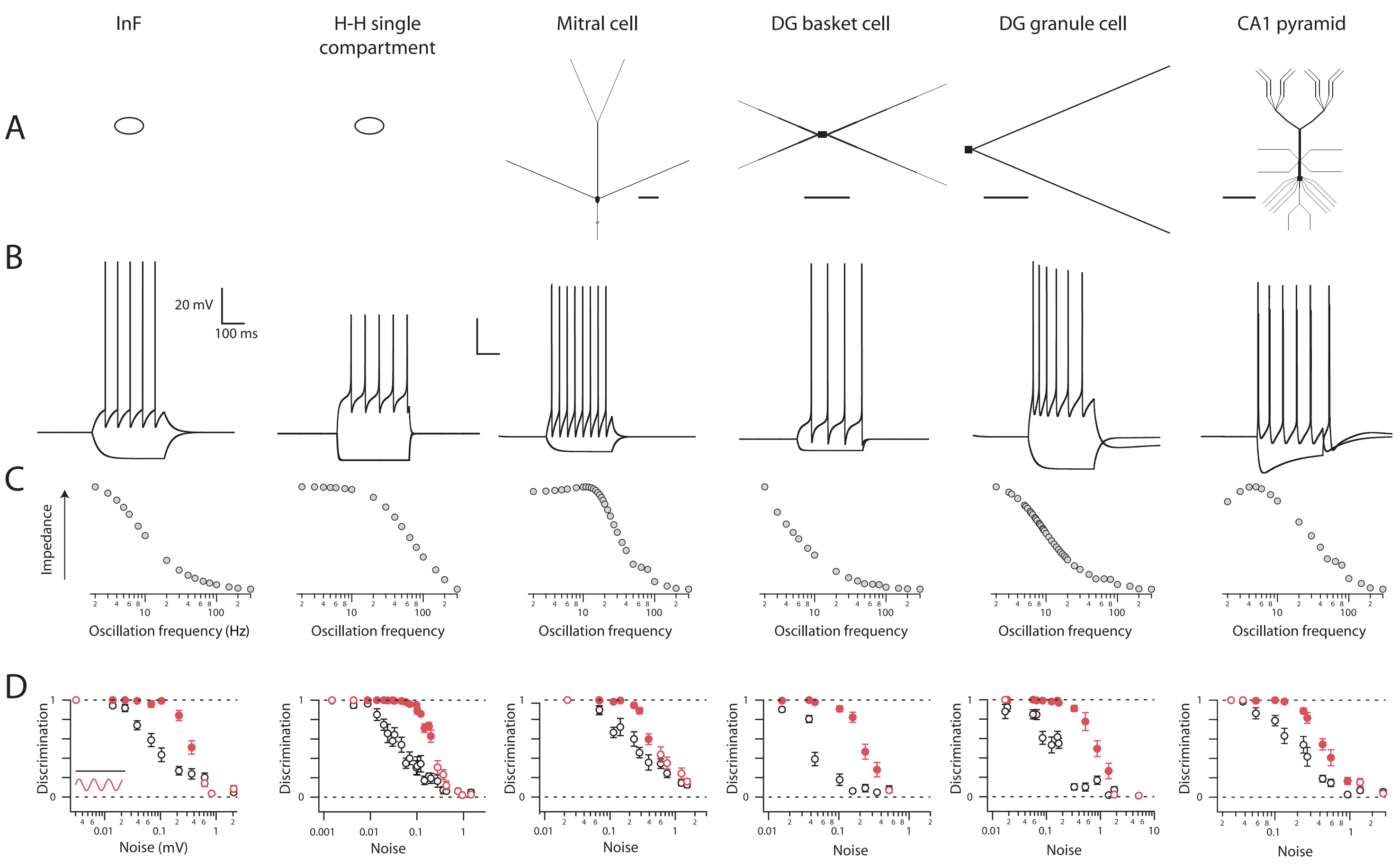

Figure S1 - Schaefer, Angelo, Spors and Margrie
